# Socialization of Gender Stereotypes Related to Attributes and Professions Among Young Spanish School-Aged Children

**DOI:** 10.3389/fpsyg.2020.00609

**Published:** 2020-04-24

**Authors:** Irene Solbes-Canales, Susana Valverde-Montesino, Pablo Herranz-Hernández

**Affiliations:** Departamento de Investigación y Psicología en Educación, Facultad de Educación, Universidad Complutense de Madrid, Madrid, Spain

**Keywords:** gender schema, professional roles, personal attributes, childhood development, environment, gender flexibility

## Abstract

Modern societies increasingly show more egalitarian attitudes related to sexism and gender equality. However, there is still an important gender gap in wages and professions as well as in expectations surrounding male and female characteristics. Developmental studies carried out from an ecological perspective confirm that these influences come from the closest environments (mainly family and school) but also from more distant systems such as media or cultural values. As children are socialized in these norms and values, they increasingly internalize those schemes and use them to judge others, to choose friends and playmates, and to construct expectations of them. On this basis, the aim of this study was to examine the degree of gender bias internalization in a group of Spanish children. Two tasks were applied to a group of 149 public school boys and girls (aged 4–9 years). Results showed that, already from an early age, the participants had internalized traditional gender roles, especially when asked to assign masculine attributes. Moreover, group differences were found given that boys seemed to be more aware of expectations surrounding masculinity and girls assigned the attributes associated with femininity to women more often than boys. Furthermore, a developmental pattern similar to one obtained in previous studies was observed. Younger children already apply gender roles as part of their increasing acquisition of knowledge in the social field, but there is a big increase in the strength of this bias as they grow older. Psychological and educational implications of these findings are discussed, especially considering that the male gender role seems to be more rigid and less malleable. In this regard, developmental and environmental studies should be considered when designing early intervention programs to reduce sexism and to promote equity in schools and families. As research has already shown what type of environments affect children’s acquisition of traditional gender roles, society must make an effort to promote more egalitarian environments that will serve as protective factors in their future psychological, social and professional development.

## Introduction

In recent decades there have been significant advances in terms of gender equality in the economic, education, and employment fields. These advances have resulted in numerous laws and regulations that seek to promote equal opportunities for men and women throughout their life and to reduce sexism in all its aspects. However, significant gender inequality in adulthood still exists in crucial areas such as wages and positions of power (vertical segregation) (Cohen and Hilgeman, 2006), as well as large differences in numerous areas of life: to give some examples, girls tend to perceive themselves as less competent in science-related fields (OECD, 2020), and women tend to be underrepresented in traditionally male professions, which are usually the ones with greater social prestige (horizontal segregation) (Cohen and Hilgeman, 2006). On a personal level, gender roles affect the physical and mental health of men and women (World Health Organization [WHO], 2002; Mayor, 2015), with gender inequality being at the base of gender violence (McCarthy et al., 2018). Moreover, although life expectancy is greater for women, they have a worse quality of life (Rollero et al., 2014).

Regarding childhood, there are multiple studies that show how boys and girls internalize the traditional gender roles that prevail in society from an early age (Jackson, 2007). This internalization has a decisive effect on their academic development, their perception of their own abilities (regardless of their real abilities), and their personal, vocational and job aspirations (Kollmayer et al., 2018).

Under these differences a persistence of traditional gender stereotypes exists, among other social factors, according to which certain attributes, behaviors and expectations are associated with men and women in a biased manner. Research in the area of developmental and social psychology has been asking for decades about the development and acquisition of gender roles in childhood, and what factors affect these ongoing events. Sex typing or the process of acquiring gender roles is a global phenomenon that children from all cultures go through, considering this as a part of regular development and a consequence of differentiated socialization processes in the first years of life. This phenomenon continues to evolve throughout life with messages that come from the contexts with which children interact (Bem, 1983; Liben and Bigler, 2002c).

Understanding how gender roles arise and are constructed has obvious practical implications. It can be useful for researchers working in the field of psychology and education or in the design of public policies when implementing real measures that promote authentic social changes in this regard (Liben and Bigler, 2002a). In addition, as these authors suggest, the study of the acquisition and development of these roles would allow us to deepen the classic nature versus nurture debate. Even when there may be small basic biological differences between men and women, the different environmental experiences we receive from the moment of birth on, as well as the interaction between both factors, determine separate paths for both.

Research in this area has proposed three major types of theories to explain gender differentiation (Liben and Bigler, 2002b). First, there are investigations that consider gender stereotypes to develop because they reflect real biological differences inherent in the two sexes (related to sex-linked genes, hormones or brain differences). Developmental psychology is framed within this approach, which states that men and women are different because human evolution has caused us to face different adaptive problems, which causes natural selection to prioritize characteristics in men and others in women (Buss, 1995). Recent studies also indicate that certain cultural values, universally associated with femininity and masculinity, could have a certain genetic origin, especially for cases where the person shows counter stereotypic values (Knafo and Spinath, 2011). Other works carried out with large samples of sibling pairs indicate that genes seem to have some weight in the development of sex-typed behavior, although the influence of the environment is very relevant (Iervolino et al., 2005).

Beyond biological theories, multiple investigations highlight precisely the role of the environment in the construction of gender roles (Carli and Bukatko, 2000). According to these approaches, men and women behave differently because of the existence of socialization practices and learning mechanisms that generate and maintain gender differentiation (Liben and Bigler, 2002b).

Here we can find the traditional learning theories, which indicate that the different environments in which children grow up reinforce and punish behaviors associated with sex, especially by significant adults (family and teachers) (Eccles et al., 2000; Beaman et al., 2006) but also by peers and equals (Witt, 2000). Social learning theories further indicate that children learn what is appropriate for their sex by observation and by imitating the behaviors of the people they identify with, who are more often of the same sex, and by observing the reactions of the environment to the models’ behaviors through a process of vicarious learning (Endendijk et al., 2018).

There is a third group of cognitive theories, which have been described as “gender constructivism.” The authors highlight the processes of individual development in the construction of gender identity and sexual roles (Bem, 1981). As a child starts to perceive that there are two types of people in social life (men and women), he/she develops a gender scheme that will guide the future processing of information from this categorization (Bem, 1981). When children understand which group they belong to and assume the stability of this situation, they begin to associate behaviors and expectations with one or the other according to their experiences, given that this scheme becomes a highly available category in social life (Bem, 1983). From this moment on, they apply gender schemes to the development of their own identity (Ruble et al., 2007), as well as to the expectations they develop regarding people with whom they interact in the attitudinal, personality or occupational spheres (Levy and Sadovsky, 2000; Halim et al., 2013). However, children are not limited to assuming and copying the gender roles that the environment provides or that biology imposes, instead they are active agents that modify the schemes as they interact with different contexts. Certain environmental experiences stimulate the restructuring of these schemes (for example, showing counter-stereotypical models) (Olsson and Martiny, 2018), which promote an accommodation of new information which results in the roles gaining a more personal content. In this line, the contributions of poststructural feminism could be framed (Renold, 2004): if gender identity were something immutable, we would limit ourselves to copying and reproducing roles as fixed schemes, which would have prevented great social advances regarding the place that men and women occupy in society.

The theories that emphasize the role of the environment in the construction of gender roles can be framed in the ecological model of Bronfenbrenner (1994), who highlights the role of the contexts with which we interact in human development and learning. Applying this model to the acquisition of gender roles, it is currently known that the socialization of gender identities and stereotypes is built upon the basis of multiple messages. These messages are often explicit (for example, a father saying to his son “boys don’t cry”), but they are also often implicit or subtle (such as underrepresentation of women in textbooks associated with science, or the low participation of men in housework). In addition, these influences come from all socialization agents (family, teachers, school, media, laws, etc.) (Shen-Miller et al., 2011; Baker et al., 2016; Paul Halpern and Perry-Jenkins, 2016).

Among the studies that analyze purely environmental variables and their effect on gender socialization processes, the negative influence of the contexts in which institutional sexual segregation is applied (Wong et al., 2018) could be highlighted. This would include, for example, the existence of educational centers segregated by sex, but also other aspects in the educational context such as the use of gender labels to form lines or the organization of classroom structures or school activities (Bigler, 1995) or basically any type of context in which the saliency of a social categorization variable (such as sex) increases the development of more rigid stereotypes (Bigler and Liben, 2007).

Considering the environmental variables that affect the development of gender roles, purely physical aspects of the environment have been studied, but also symbolic and discursive. Within the school, the use of spaces has been analyzed profusely, highlighting how in general boys tend to make greater use of public spaces (playgrounds or even hallways) with games that displace other activities (Clark and Paechter, 2007), compared with girls, who tend to make smaller groups and relegate to private spaces (Børve and Børve, 2017). Along these lines, some reviews highlight how the classroom is not only a context in which interactions occur, but rather that it reproduces and is in turn produced by the inequalities of power that exist in society (Shilling, 1991). The distribution of the classroom, objects and spaces within a school seem to reproduce gender differences, although they sometimes leave room for more flexible non-normative discourses (Lyttleton-Smith, 2019). On a more symbolic level, the school context also transmits gender stereotypes through the so-called hidden curriculum, which would include subtle and implicit messages, in some unconscious cases, about situations of power and subordination, what is expected of each child in function of their social origin, or ethnicity, as well as whether they are a boy or a girl (Basow, 2004).

Differentiated environments for children are also observed in the family, even before birth. Different studies show how different colors are used in children’s rooms, including different types of objects, decorations and toys which highlight the performative nature of the use of the space (Pomerleau et al., 1990). Furthermore, family contexts where sexual differentiation of tasks is more traditional influence the child’s acquisition of gender stereotypes (Paul Halpern and Perry-Jenkins, 2016).

The power of the messages included in children’s popular culture, including television, series or internet, which children and adolescents seem to consume increasingly despite reproducing evident forms of sexist messages and gender stereotypes, should also be considered when talking about environmental influences (Aubrey and Harrison, 2004; Döring and Mohseni, 2019).

From all these environmental influences, during their first years of life, children construct the gender schemes that will guide the elaboration of expectations about what society expects of men and women. In developmental psychology, the process of acquiring these schemes is called sex typing (Bem, 1981), and implies the application of gender stereotypes to multiple areas that range from material aspects that are differentially associated with one group or another (colors, toys, or objects), to complex social constructions such as expectations in regard to personality, skills, or professions that men and women carry out (Jackson, 2007; Wilbourn and Kee, 2010; Patterson, 2012). These stereotypes involve the development of differentiated schemes associated with masculinity and femininity (Liben and Bigler, 2002c), which interact with the child’s own sex as he/she is building them. This causes an earlier acquisition of stereotypes associated with men by boys, and with women by girls, giving priority to those that are most useful for building their own identity (Bem, 1981; Liben and Bigler, 2002c).

Developmental studies that have been carried out on this subject therefore seem to indicate that we are faced with a multidimensional construct that is acquired gradually (Liben and Bigler, 2002c; Halim et al., 2017), in interaction with the physical and symbolic environments that surround us, whose acquisition also influences cognitive (mainly flexibility and multiple categorization abilities) and motivational aspects of children (Bem, 1981; Bigler, 1995; Weisgram, 2016; Halim et al., 2017). Around the age of 3, children seem to clearly understand that the world is divided between men and women, and that they belong to one of those categories. From the moment in which they acquire the notion of gender constancy (Ruble et al., 2007), they identify with one of the groups and begin to fill these categories with information, tending to prefer one’s own group over the foreign group, attributing positive aspects to the in-group over the out-group and preferring peers over those who belong to the other category (Halim et al., 2017). Thus, what some authors call gender rigidity appears (Halim, 2016), which leads to gender differentiation to become especially strict during these years. Children begin to progressively associate professions, skills and objects in a biased way in line with the cultural knowledge they have absorbed (Jackson, 2007; Bian et al., 2017). The phenomenon of gender typing usually progresses throughout the primary school stage (6–11 years), when the stiffness of the traditional roles that apply to themselves and the rest begins to decrease (Trautner et al., 2005; Ruble et al., 2007; Siyanova-Chanturia et al., 2015), due to an increase in cognitive flexibility, among others (Bigler, 1995; Banse et al., 2010). From this moment on, if cognitive progress continues and learning environments are sufficiently egalitarian, stereotypes tend to become more flexible and roles blur (Bennett and Sani, 2006; Halim, 2016). However, as is obvious, in many cases stereotypes also persist throughout life and continue to influence the behavior of adolescents and adults.

The developmental pattern described has been confirmed in multiple investigations that have been carried out in recent decades with children from different cultures, although as mentioned before there are differences in the developmental course of the different components associated with gender schemes, as this is a multifaceted construct (personal attributes, professions, skills, objects, etc.). It seems that gender biases tend to be more congruent in their multiple facets as the child’s age progresses (Liben and Bigler, 2002c). In addition, the developmental course varies significantly when we talk about aspects associated with masculinity, compared to the characteristics that are usually associated with femininity. The data seem to indicate that, in a general way, the professions, objects or attributes associated with men tend to be more rigid, punishing more radically the behaviors that transgress gender mandates for men in some way (Wilbourn and Kee, 2010).

In this sense, an asymmetry of gender stereotypes exists: gender stereotyping is less restrictive for female stereotypes than for male stereotypes (Wilbourn and Kee, 2010; Siyanova-Chanturia et al., 2015). In addition, several studies indicate that girls generally show more flexible gender stereotypes than boys (Shen-Miller et al., 2011; Siyanova-Chanturia et al., 2012), especially in the area of domestic activities (Poulin-Dubois et al., 2002).

Furthermore, there are important differences in the development of gender differentiation between boys and girls, undoubtedly related to the social position they occupy. For example, both groups tend to associate positive characteristics preferentially with their own group, but after the age of 6 girls stop showing this pattern and mostly consider that something that requires a lot of intelligence should preferably be done by a man (Bian et al., 2017).

Based on these previous findings, the objective of this study is to analyze the presence of gender schemes in a group of Spanish children between 4 and 9 years of age, being as far as we know, the first study conducted on this topic with a children’s sample in our country, a country which has historically been dominated by a macho culture that has evolved in recent years toward more egalitarian attitudes (López-Sáez et al., 2008). Although some researches have been done on the topic with Spanish adolescents and young adults (Colás Bravo and Villaciervos Moreno, 2007; Ferrer-Pérez and Bosch-Fiol, 2014), none of them have focused on early ages, where the origin of the problem is located, using a developmental approach. The results of the study might be helpful when designing educational and policy interventions in order to eliminate gender stereotyping as soon as possible, before those social shared schemes have been irrevocably internalized by the children.

## Materials and Methods

### Participants

The participants were school children from a public primary school in the Community of Madrid in an area of medium socioeconomic status. After the acceptance of the school’s management team regarding participation in the study, an informative document with an authorization was sent to the families of students between the ages of 4 and 9. Ultimately, 149 children participated in the study and their ages ranged from 4 to 9 years (*M* = 6.25; *SD* = 1.38), distributed in three age ranges. A first interval included 4- and 5-year-old participants and consisted of 22 boys and 27 girls. The next interval covered the range of 6- and 7-year-olds and consisted of 40 boys and 27 girls. The third interval, the 8- and 9-year-olds, included 16 boys and 17 girls.

### Materials

Two types of tasks were developed specifically for this study: Task 1, aimed at assessing stereotypes related to typically female or male personal attributes, and Task 2, designed to identify stereotypes related to professional roles. Supplementary Material includes the instructions used to apply both tasks.

#### Task 1: Personal Attributes

The personal attributes selected for this study were: smart, kind, aggressive, vain, happy and grumpy. These attributes were chosen from the Bem Sex Role Inventory (BSRI) (Bem, 1974), including a positive and a negative attribute for each category, as well as characteristics that could be understood by the children of these ages. According to this instrument, the smart and aggressive attributes are stereotypically masculine adjectives, while the kind and vain attributes are typically feminine. The happy and grumpy attributes would be considered neutral (they are not culturally associated with either the male or the female gender).

The procedure for applying the task was based on the one used in a recent study that had similar objectives (2). Each participant was told six stories in which the protagonist was a very smart, kind, aggressive, vain, happy or grumpy person. This task had two versions: one in which the protagonist was an adult (man or woman) and another in which the protagonist was a child (boy or girl). The participants had to choose, in different tests, between four images of adults (two women and two men) and four images of children (two boys and two girls), who they considered the protagonist of the different stories was. The stories are described in more detail in the next section.

The photographs of men, women, boys, and girls used for the smart and kind attributes were taken, with prior consent of the authors, from the study carried out by Bian et al. (2017). To select the photographs corresponding to the rest of the personal attributes, a previous study was carried out, in order to homogenize the level of physical attractiveness of the men and women that appeared in the photographs, so that this characteristic did not bias the participants’ responses. To do this, 16 photos of men, 16 of women, 16 of boys and 16 of girls were located in a databases of free-use photographs. All subjects were approximately the same age, appeared in the photograph only in portrait format (mainly the face and some of the upper body) and were smiling. The photos were included in a questionnaire applied through the Google Form tool to 55 adults, who were asked to rate the level of physical attractiveness of each person from 1 to 4. Of the 64 photographs included in the previous study, 32 were selected for this study: 8 photographs of men, 8 of women, 8 of boys and 8 of girls. The selected photographs were matched (men with women on the one hand, and boys with girls on the other) considering the means of each person’s level of attractiveness. These photographs were added to the previous 16, so a total of 48 photographs distributed in 12 tests were finally used (6 with adult photos and 6 with children photos). The 48 cards with photographs had dimensions of 9 × 6 cm. For each attribute, 4 cards were presented (for the adult attributes version, 2 photographs of men and 2 photographs of women; for the children attributes version, 2 photographs of boys and 2 of girls). With this task, three different scores were calculated:

*Male roles attributed to men* measured the degree of stereotyping of male attributes. To calculate the corresponding score, each time a participant chose the photograph that corresponded to the stereotype, it was scored with a 1. For example, if a characteristic stereotypically attributed to men, such as aggressive, was being assessed, and the participant attributed that feature to the photo of a man or a boy, it was assigned a score of 1 in that test. Subsequently, a proportion of the biased responses on the total male attributes, which ranged from 0 to 1, was calculated in order to compare the scores obtained in all tasks on the same scale.

*Female roles attributed to women* measured the degree of stereotyping of female attributes. For its calculation, a criterion similar to that previously mentioned was followed, but in this case in relation to female attributes.

*Stereotyped roles attributed to men and women* measured the degree of global stereotyping with respect to gender, applied to men and women as a whole. In this case, the score was also calculated proportionally at a value of 0 to 1, which summarizes the degree to which the participants apply the gender scheme when assigning attributes associated with masculinity and femininity.

#### Task 2: Professional Roles

Task 2 is adapted from the professional role attribution instrument included in the work of Liben and Bigler (2002f). The task was to show an image related to a profession and ask who should carry out that profession, giving the option of answering “only women,” “only men,” or “both.” The selected professions considered in this study represented four typically masculine jobs (police, ship captain, scientist, and computer specialist), four typically feminine (nurse, cashier, florist, and hairdresser) and two neutral (artist and baker). To support the application of this task, 10 rectangular cards were used, measuring 10 × 11 cm. Each card contained a representative drawing of the professions with objects associated with them (for example, a bouquet of flowers for the florist profession). Three rectangular cards, 18 × 14.5 cm, were also used which served as visual support for the three response options. For each profession, the participants were asked who they thought should do each job, giving them the option to answer “only women” (card with a woman’s silhouette), “only men” (card with a man’s silhouette), or “both” (card with the silhouette of a man and a woman together). Information on the following variables was obtained with the administration of this task.

*Male professions attributed to men* measured the degree of stereotyping of masculinized professions (police, ship captain, scientist and computer specialist).

*Female professions attributed to women* measured the degree of stereotyping of feminized professions (nurse, cashier, florist and hairdresser).

*Stereotyped professions attributed to men and women* measured the degree of global stereotyping regarding gender in the professional domain, applied to men and women as a whole. In this case, the score was also calculated proportionally at a value of 0 to 1, which summarizes the degree to which the participants apply gender schemes when giving their opinion about who should perform different types of strongly stereotyped professions.

To codify these variables, the criteria proposed by the creators of the measure (4) were followed. The scores were obtained by calculating the proportion of stereotyped responses in each case. That is, the proportional number of responses in which the items of male professions were assigned to the category “men only” was considered, as well as the proportion of items of female professions assigned to “women only,” obtaining final scores of 0 to 1. Higher values in these variables indicate greater gender stereotyping.

To complement the results of the stereotyped responses observed in this task, several measures that represent the degree of flexibility when applying gender schemes to professions were also calculated following Liben and Bigler (2002f). Higher values indicate greater flexibility in the profession’s field regarding gender roles. Thus, proportional scores (with values from 0 to 1) were recalculated for three specific areas:

*Flexibility male professions* measured the degree of flexibility of typically male professions. For the response to be considered flexible, the subject had to choose the option “both men and women” in the specific items.

*Flexibility female professions* measured the degree of flexibility with respect to professions considered “feminine.” As in the previous variable, in order for the response to be considered an indicator of flexibility, the subject had to choose the option of “both men and women” with respect to professions considered typically feminine.

*Global flexibility* measured the combined degree of flexibility, both for typically male and typically female professions.

### Procedure

The participants performed the tasks in classrooms of their school that met the necessary conditions of space, silence, and luminosity to conduct the interviews and outside of their usual school hours. In the task related to personal attributes, the application procedure was similar to the one applied in the original study by Bian et al. (2017).

For each attribute, a story was told in which the protagonist stood out because of this specific attribute. Subsequently, the subject was asked to select, from four options, the photograph of the person who he/she considered that could be the protagonist of that situation. When the tests were conducted in Spanish, gender neutral terms were used, such as: “a person,” or “someone” avoiding biasing the answers with the questions asked. For example, one of the stories explains: “*In the place where I work there are many people. But there is one particular person who is different. That person is very, very vain. This person looks constantly in the mirror and worries about whether their hair and clothes look good. This person is quite vain. Who do you think, out of these 4 people, is the vain person from the story?*”. Four different photos were then placed on the table for each attribute (2 men and 2 women in the adult version/2 boys and 2 girls in the children’s version). When the participants pointed to one of the photos, the response was recorded, and the next story began. This continued in the same way until all the attributes of the adult version were completed, and then all those corresponding to the children’s version. In both versions, the traits evaluated were the same, using stories adapted for adulthood and childhood, in the same order of presentation. In each test the four photographs were presented randomly for each participant and for each attribute. The possibility of selecting the “don’t know” option was offered when the participant could not decide between the different people, although this response was only sporadically used by 5 of the participants in regard to some specific attribute. These cases have been coded as “lost cases” for those specific attributes.

Regarding the procedure for applying task 2, the same procedure proposed by Liben and Bigler (2002f) was applied, accompanied by the visual support cards. Before presenting the professions, the three cards with the silhouettes of men and women were placed on the table, placing the card that indicated that “both” could carry out each of the professions in the center, and the other two randomly to the right and to the left of the participants. In this case, participants were explained that they would be presented with different cards with drawings related to different professions. The task was to decide if they considered that this profession should be carried out only by men, only by women, or by both, by placing the card of each profession on the table space occupied by the silhouettes already described. The order of appearance of the cards was as follows: nurse, police, cashier, artist, ship captain, florist, scientist, baker, hairdresser, computer specialist, interspersing typically male, female and neutral professions randomly. For example, for the hairdressing profession, a card with an image of a comb and scissors was shown and the following was said: “*Who do you think should be the person who cuts your hair when you go to the hairdresser? Is it a profession that only men should do, only women, or that both should?*” In each test, the order of presentation of “men only” and “women only” in the instructions was varied, so that the order of presentation of the response options did not bias the results.

## Results

### Descriptive Statistics of Measures

Table 1 presents the proportion of tests in which the participants assigned both male and female attributes to the two types of targets, including in all cases the photos of adults and those of children as a whole. As can be seen, the attributes considered as masculine were associated more frequently with men than with women, this difference being significant [*t*(145) = 7.07, *p* = 0.00]. On the other hand, the attributes considered feminine were attributed more to women than to men [*t*(144) = 4.51, *p* = 0.00]. Considering the attribution of stereotyped responses globally in this task (Stereotyped attributes – total score), the value obtained in this variable indicates that biased attributes were assigned to the target gender in more than 60% of the tests.

**TABLE 1:** Proportion of masculine/feminine attributes assigned to male/female targets in Task 1.

		***M (SD)***
Masculine attributes	*Male targets*	0.66 (0.28)
	*Female targets*	0.34 (0.28)
Feminine attributes	*Male targets*	0.39 (0.29)
	*Female targets*	0.61 (0.29)
Stereotyped attributes (total score)		0.63 (0.19)

*Minimum score = 0; maximum score = 1.*

To test whether there was a greater stereotyping of male or female roles, the *t*-test was applied for related samples, confirming that there were no significant differences in both types of stereotyping (*p* = 0.16), although the mean was slightly higher for the stereotyping of masculine attributes.

The data therefore confirmed the biased assignment of personal attributes to unknown people, both adults and children. In terms of the specific individual attributes, the smart attribute was the most skewed attribute in its assignment, being mostly associated with men (*M* = 0.70) versus women (*M* = 0.30) [*t*(146) = 6.88, *p* = 0.00]. Aggression was also preferentially assigned to male targets (*M* = 0.62) versus female targets (*M* = 0.38) [*t*(146) = 3.75, *p* = 0.00]. Regarding the attributes considered feminine, being vain was the attribute most frequently associated with women (*M* = 0.73) versus men (*M* = 0.27) [*t*(146) = −7.95, *p* = 0.00]. However, being kind was assigned to men and women to the same extent (*M* = 0.51 and 0.49, respectively).

Table 2 summarizes the scores regarding the assignment of professions to men, women, or both. As can be seen, the average of the responses that indicated that stereotyped jobs should be carried out by both sexes reached a high value (*M* = 0.45), with it being the most frequent type of response. This score indicates a remarkable flexibility in the professional area.

**TABLE 2:** Proportion of masculine/feminine professions assigned to male/female targets and flexibility scores in Task 2.

		***M (SD)***
Masculine professions	*Only men*	0.51 (0.30)
	*Only women*	0.09 (0.16)
Feminine professions	*Only men*	0.12 (0.18)
	*Only women*	0.38 (0.27)
Stereotyped professions (total score)		0.45 (0.25)
Flexibility for masculine professions		0.40 (0.30)
Flexibility for feminine professions		0.50 (0.29)
Flexibility (total score)		0.45 (0.25)

*Minimum score = 0; maximum = 1.*

Analyzing only the responses regarding male professions, these were assigned to a much greater extent only to men than to women [*t*(148) = 14.21, *p* = 0.00]. For their part, professions considered feminine tended to be considered as something that only women should do in many more cases than something that only men should do [*t*(148) = −8.53, *p* = 0.00]. In order to verify if there was a greater application of gender schemes in the domain of male or female professions, the *t*-test was applied for related samples, observing significant differences between the average for male professions assigned to men and the average for female professions assigned to women [*t*(148) = 5.42, *p* = 0.00]. This result is confirmed by comparing flexibility measures for male and female professions, with less flexible responses for male professions than for female professions [*t*(148) = −4.12, *p* = 0.00].

**FIGURE 1:**
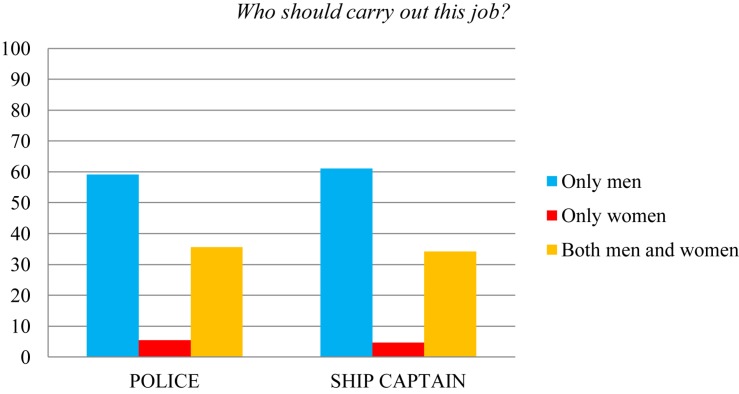
Percentages of assignment of the police and ship captain professions.

**FIGURE 2:**
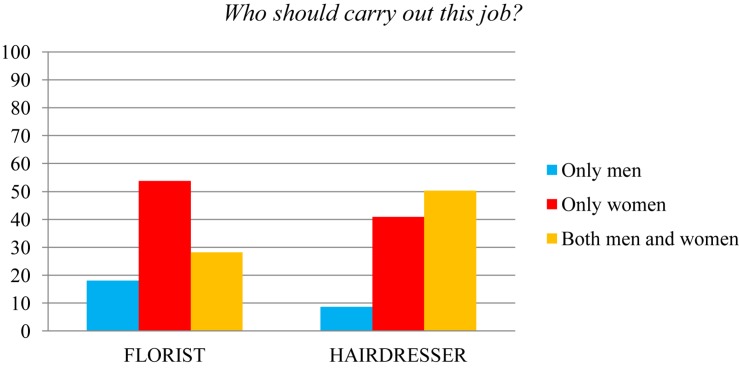
Percentages of assignment of the florist and hairdresser professions.

Diving in to a more precise analysis of the specific professions included in this study, the most biased professions that can be observed in the case of the male gender (see Figure 1) were those of police (59.1% of the participants thought that “only men” should exercise this profession) and ship captain (61.1% of restrictive responses for men). On the other hand, the professions most linked to women (see Figure 2) were those of florist (53.7% of stereotyped responses) and hairdresser (40.9%).

### Gender Differences

Table 3 shows the information related to gender differences in all the variables of interest. To simplify the analysis of the results and given that the attribute scores assigned to masculine targets and feminine targets in each case are complementary, only the stereotyped attributes of each personal attribute are presented. The results indicate that boys stereotype more male attributes by assigning them more to boys than girls [*t*(129.07) = 3.63, *p* = 0.00]. On the other hand, girls seem to internalize the attributes associated with femininity more intensely than boys [*t*(143) = −3.77, *p* = 0.00]. Furthermore, the total score regarding stereotyped attributes (which includes male and female attributes assigned in a manner consistent with gender schemes) does not show differences in either group (*p* = 0.68).

**TABLE 3:** Descriptive statistics for boys and girls.

	***M* (*SD*)**	
	**Boys (*n* = 78)**	**Girls (*n* = 71)**
Masculine attributes assigned to male targets	0.74 (0.24)	0.58 (0.30)
Feminine attributes assigned to female targets	0.53 (0.29)	0.70 (0.28)
Stereotyped attributes (total score)	0.63 (0.17)	0.64 (0.20)
Masculine professions assigned to “only men”	0.53 (0.32)	0.50 (0.27)
Feminine professions assigned to “only women”	0.34 (0.29)	0.42 (0.25)
Flexibility for masculine professions	0.40 (0.32)	0.40 (0.27)
Flexibility for feminine professions	0.51 (0.30)	0.49 (0.26)
Flexibility (total score)	0.45 (0.27)	0.44 (0.22)

*Minimum score = 0, maximum = 1.*

Regarding professions, boys and girls stereotype traditionally masculine professions to the same extent (*p* = 0.65). In the case of the attribution of female professions assigned only to women, this is a more common response among girls than among boys, although the differences only reached a level close to statistical significance (*p* = 0.09). Flexibility when assigning stereotyped professions, does not differ between boys and girls when analyzed together (*p* = 0.78), nor when masculine or feminine professions are analyzed separately (*p* = 0.93 and *p* = 0.57, respectively).

### Age Group Differences

Table 4 shows the results for the three age groups. The results of the ANOVA, applied to compare the statistics of the three groups, are also included in the table, in addition to the corresponding *post hoc* test when significant age differences were found. As can be seen in the table, significant differences appear in the stereotyped assignment of male attributes to men. Specifically, the internalization of these schemes seems to increase with age, being only the differences between the youngest and oldest group significant. Regarding the other two scores related to personal attributes, there are no significant differences between the three age groups, the attribution of female roles to women and the total stereotyping score of personality attributes being stable. The univariate analysis does not yield significant interactions between gender and age group for any of the variables related to Task 1.

**TABLE 4:** Descriptive statistics for the three age-groups.

	**ANOVA**						
	***M* (*SD*)**			***F***	***DF***	***p*-Value**	***Games-Howell***
	**Group A 4- and 5-year-olds (*n* = 49)**	**Group B 6- and 7-year-olds (*n* = 67)**	**Group C 8- and 9-year-olds (*n* = 33)**				
Masculine attributes assigned to male targets	0.59 (0.32)	0.65 (0.26)	0.78 (0.18)	5.07	2,143	0.007	C > A** C > B**
Feminine attributes assigned to female targets	0.61 (0.32)	0.61 (0.30)	0.60 (0.25)	0.007	2,142	0.993	–
Stereotyped attributes (total score)	0.60 (0.19)	0.63 (0.19)	0.69 (0.16)	2.41	2,141	0.093	–
Masculine professions assigned to “only men”	0.54 (0.29)	0.51 (0.31)	0.48 (0.29)	0.287		0.287	–
Feminine professions assigned to “only women”	0.46 (0.25)	0.37 (0.27)	0.26 (0.25)	6.20	2,146	0.003	C < A**
Flexibility for masculine professions	0.31 (0.26)	0.42 (0.31)	0.49 (0.29)	4.39	2,146	0.014	C > A*
Flexibility for feminine professions	0.37 (0.24)	0.54 (0.29)	0.62 (0.28)	9.84	2,146	0.000	C > A*** B > A**
Flexibility (total score)	0.34 (0.19)	0.48 (0.25)	0.56 (0.26)	9.37	2,146	0.000	C > A*** B > A**

*Minimum score = 0, maximum = 1; ^∗^*p* < 0.05; ^∗∗^*p* < 0.01; ^∗∗∗^*p* < 0.001.*

**FIGURE 3:**
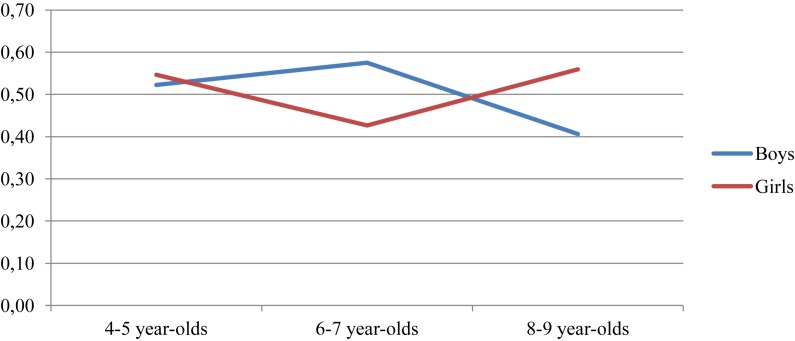
Proportion of masculine professions assigned to “only men,” by gender and age group.

Regarding the assignment of male professions to men, there are no significant differences between the different age groups. A univariate analysis of variance, including age and gender subgroups, was performed to analyze the possible interaction between these two variables. The results of this test show significant differences in this variable for the interaction between gender and age group [*F*(2,148 = 3.089, *p* < 0.05], observing that stereotypes regarding male professions increase with age in girls, but decreases among boys (see Figure 3).

**FIGURE 4:**
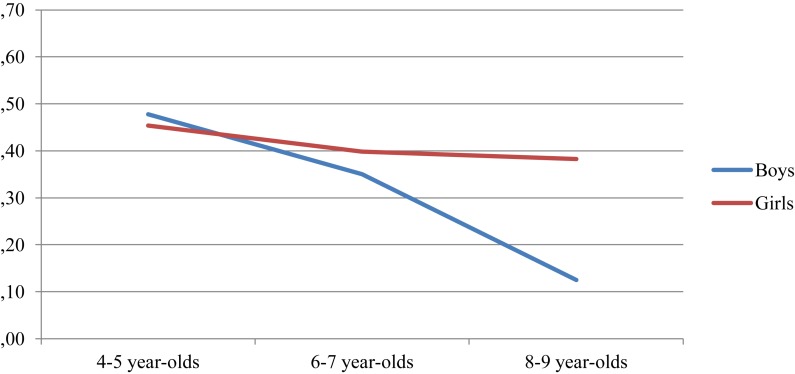
Proportion of feminine professions assigned to “only women,” by gender and age group.

Regarding the allocation of female professions only to women, there is a gradual reduction in this type of response as the participants’ age progresses. However, the corresponding *post hoc* test indicates that the differences between groups turn out to be significant only between the youngest and the in-between children, on the one hand, compared to the oldest. The univariate analysis again indicates that there is a significant interaction between the gender and the course of the participants age in relation to this score [*F*(2,148) = 3.069, *p* = 0.05]. As can be seen in Figure 4, the girls’ scores hardly vary with age, while the boys’ scores fall drastically in the group of the oldest children, with the percentage of boys of these ages who consider that these professions should be carried out only by women being very small.

Regarding the scores related to the flexibility in the professions task, the results of the ANOVA showed significant differences between the age groups in the global flexibility score, in line with the other variables obtained from this task [*F*(2,148) = 9.377, *p* = 0.00]. An increase in flexibility is observed as the age of the participants increases (especially among boys). The differences are significant both for the scores regarding the flexibility of typically male professions [*F*(2,148) = 4.397, *p* < 0.05], and for typically female professions [*F*(2,148) = 9.841, *p* = 0.00]. *Post hoc* tests indicate, as can be seen in the table, that the differences between the age groups were significant between the youngest and the oldest (flexibility for male professions) and between the youngest and the other two groups (flexibility for female professions).

### Correlational Results

A correlational analysis of all the scores calculated in Tasks 1 and 2 was performed. The results indicate that, in general, there is only a significant correlation between the measures: positive relationship between the variables Masculine professions assigned to “only men” and Feminine professions assigned to “only women” (*r* = 0.39, *p* = 0.00). This result shows that participants who stereotype the professions considered more typically male tend to do the same for professions usually considered female. For the rest of the scores, there is no significant relationship between the variables, which reveals that these are independent variables when all participants are considered as a group.

When the sample is divided into different groups and the sociodemographic variables (gender and age group) considered in this study are included in the analysis, interesting results appear in the specific correlations of the different subgroups. Thus, the correlation between the stereotyping of male and female professions turns out to be significant again in the boys’ and girls’ groups, considering them separately, but more intense among boys (*r* = 0.461, *p* = 0.00) than among girls (*r* = 0.311, *p* = 0.008).

On the other hand, dividing the sample into the three age groups that have participated in the study, no significant correlations are found between any of the measures included in the study for the youngest group. For the in-between group, the relationship between the variable Masculine professions assigned to “only men” and Feminine professions assigned to “only women” once again reaches an important value (*r* = 0.430, *p* = 0.00), with correlation values increasing between the oldest group (*r* = 0.501, *p* = 0.003).

Moreover, in the group of older participants, significant relationships are observed among other variables. Thus, the participants who stereotype the supposedly masculine personal attributes are also the ones that stereotype the male professions most rigidly (*r* = 0.378, *p* < 0.05) and therefore show lower levels of flexibility in this type of tasks (*r* = −0.360, *p* < 0.05). This relationship is not observed for the relationship between personal attributes associated with femininity and the responses of participants in the field of feminized professions.

## Discussion

The results confirmed that the children between 4 and 9 years of age who took part in our study have generally internalized gender schemes, which they clearly make use of when assigning personal attributes or professional roles. However, these general results have slight variations and different developmental trajectories between the boy and girl groups, as well as in the relationship to masculinity and femininity, following the line of multiple previous studies carried out in this area (Levy and Sadovsky, 2000; Liben and Bigler, 2002b; Jackson, 2007; Miller et al., 2009; Wilbourn and Kee, 2010; Halim et al., 2017). As it will be thoroughly described above, most of the findings of the present study are similar to the ones obtained in previous researches with children in another countries, although the heterogeneity of measures makes difficult in some cases to obtain clear conclusions about the generalization of them.

### Stereotyping of Personal Attributes

As recent previous studies have shown (Liben and Bigler, 2002b; Siyanova-Chanturia et al., 2012, 2015), boys tend to produce biased responses more frequently when assigning male traits, while girls tend to issue stereotyped responses more frequently for female traits. This indicates that boys and girls pay more attention to the traits associated with their own group. They internalize them earlier and incorporate them as more rigid schemes when it comes to creating expectations regarding unknown people. Given that the construction of this scheme occurs in parallel to gender identity development, it is more efficient for girls, from an adaptive point of view, to pay attention to what society expects of them because they are women. This acquisition allows them to incorporate these expectations into their own identity, in the same way that happens to boys. Incorporating specific gender roles at such early ages (for example, associating aggressiveness with masculinity and lower intelligence to women), in parallel to the development of gender identity, is something that can have obvious implications for children as it adds constraints to their development at such an early stage (Bem, 1981; Brinkman et al., 2014; Kurtz-Costes et al., 2014).

Moreover, the biased responses in the assignment of personal attributes were more frequent for male attributes, although the difference does not have a sufficient level of significance. However, previous research done in this area confirms that gender roles are generally more rigid for men than for women, so we can confirm that this greater lack of flexibility associated with masculinity is already perceived and internalized by our participants in the first years of life (Wilbourn and Kee, 2010; Siyanova-Chanturia et al., 2012). Nevertheless, it must be noted that other studies have found the opposite result (Halim et al., 2014; Baker et al., 2016).

The fact that in a very high proportion of the tests (almost three quarters of them) intelligence was assigned to boys has undeniable consequences for the development of girls, as described in other previous studies with similar results (Bian et al., 2017). If girls perceive that very intelligent people are usually men, they will tend to apply that association to their own self-concept and will project expectations aligned with this association which reinforces their low presence in STEM careers, as well as a worse self-perception of personal skills to face general problems, because intelligence is a necessary attribute for all areas of life. Data obtained in multiple studies indicate that these types of expectations often function as a self-fulfilling prophecy, especially in situations in which girls feel that they are being evaluated, which reduces their performance in objective evaluation tests (Jussim et al., 1996; Neuburger et al., 2012; Plante et al., 2013). The educational and environmental interventions that are carried out to reduce the gender gap in the vocational and professional aspirations of children and young people should undoubtedly use this information to design effective strategies from an early age which draw on a thorough analysis of their expectations and dismantle this type of bias that is so limiting for women’s aspirations.

It should also be noted that an important part of the responses indicate that aggressiveness is also a trait strongly associated with masculinity, as found in previous studies (Baker et al., 2016). This seems to confirm that children perceive, from a very young age, that men tend to be more aggressive than women, a characteristic that can undoubtedly be found at the root of phenomena such as gender violence. Although in this study the participants have been asked to generate expectations about the presented targets (appropriation of culturally shared roles) and have not explicitly been asked whether these types of behaviors are adequate, the data show that from an early age children perceive this behavior as an attribute more associated with normative masculinity, with the implications that this has for the socio-emotional development of both groups. Boys seem to assume early on that aggressiveness is more frequent among their peers and male adults, while girls also perceive that difference, which they may find inevitable. It is convenient to consider this perception when designing strategies to prevent gender violence and any form of sexual abuse, taking away the normality surrounding this issue and teaching children that it is a cultural aspect that can be avoided. The educational objective in these cases will be to provide boys with alternative strategies to manage conflicts and to promote in all children a critical analysis of media messages that often idealize violence associated with masculinity compared to other forms of solving non-violent problems, such as negotiation or cooperation.

Regarding women, they seem to be perceived by the participants as much more concerned about their physical appearance than men. If girls internalize early on that women naturally care a lot about their image, they are more likely to feel insecure with their physical appearance and develop a more negative body image by comparing how they look with prevailing beauty canons. This aspect is at the base of various mental health problems such as eating disorders, much more frequent among women than among men (Baker et al., 2016). In this regard, learning environments should foster a more polyhedral image of women, cultivating the development of skills that are not focused on physical appearance and taking importance away from their looks. Contexts that stimulate the development of skills and competencies in all areas and foster a body experience based on enjoyment and personal acceptance (for example, through participation in sports or physical activities) will foster a positive experience and care of the body that goes beyond the socially established beauty canon.

### Stereotyping of Professional Roles

Regarding the application of gender schemes to the analyzed professions, it should be noted that an important part of the participants considered that the professions presented should be carried out interchangeably by men and women (Liben and Bigler, 2002d). This result seems to be related to the multidimensionality of the development of gender schemes (Liben and Bigler, 2002b, e), noting that the application of these schemes may vary depending on the domain in which they are applied, and the type of response options presented.

However, the data show the application of a non-negligible amount of traditional gender stereotypes when assigning professions. As in the task of personal attributes, there is a greater stereotyping of the male professions than of the female professions, again confirming the appearance of a gender asymmetry (Wilbourn and Kee, 2010; Siyanova-Chanturia et al., 2015). Consistently, the responses that indicated greater flexibility (“both men and women can carry out this profession”) were more frequent for professions associated with women than for those associated with men. The professional field seems to be, as the personal one, more rigid with respect to masculine-related schemes than with those associated with traditional feminine schemes. Thus, the more traditionally masculinized professions were more frequently “banned” for women than womanized professions were for men (for example, almost 60% of the participants considered that the police profession should be carried out only by men).

On the other hand, although the participants also applied gender biases when analyzing female professions (for example, more than half of the responses reported that the florist profession should be carried out only by women), the girls were slightly more rigid than boys when considering such professions. The tendency to perceive and internalize to a greater extent the roles attributed to one’s own group seems to be confirmed in the professional sphere only for girls, but not for boys. In short, all (boys and girls) know and moderately internalize gender schemes for male professions. However, girls seem to acquire the professional biases associated with their own sex more strongly (Baker et al., 2016).

It is interesting to note that a very important part of the participants considered that the profession of police or ship captain should only be carried out by men. This shows that although there are currently frequent contra-stereotypical examples in the workplace, there are still professional areas that are generally associated with men (Cohen and Hilgeman, 2006), in which, for children in this age range, women do not seem to have a place. The exposure of more contra-stereotypical models seems necessary when presenting examples that destroy these rigid schemes (Olsson and Martiny, 2018), since in these two professions it is certainly less frequent to find women. If early on children look at different examples of people who carry out a number of different jobs in society, regardless of their sex, they will internalize a greater flexibility as something natural that gradually destroys the horizontal gap that persists in the workplace.

It should be considered here that, as found in previous studies (Vervecken and Hannover, 2015), the professions with greater social prestige are those that are most associated with men, compared to those who receive less salary and have less status, in which case the answers are frequently more flexible. In this sense, the contexts surrounding the child (whether immediate, such as school or family, or virtual ones such as television or internet) must make an effort to destroy this rigid stereotyping of schemes when considering a specific profession such as typical of men or women. The choice of a job must be associated with the personal interests and real abilities of each person, without limiting the professional expectations of children and affecting their vocational choices on the basis of sex.

### Gender Schemes Development

Regarding the pattern observed in the development of gender schemes, the results show relevant developmental differences in some of the measures analyzed, but not all, following the results of previous studies (Liben and Bigler, 2002b; Martin and Ruble, 2004; Trautner et al., 2005; Bennett and Sani, 2006; Halim, 2016; Halim et al., 2017). Although traditional gender schemes appear to be incorporated in the youngest group (both for male and female attributes), their application of expectations regarding unknown people increases significantly with age for male roles, while in the case of women it remains stable. This indicates that the asymmetry observed with respect to male and female schemes would not yet be present at 4 and 5 years of age. However, already at 8 and 9 years of age the masculine scheme (associated with intelligence and aggressiveness) seems to be more incorporated than the feminine scheme.

The data indicate that, as of the age of 8, children have already perceived the asymmetry regarding the gender mandates previously mentioned (greater social pressure regarding the characteristics associated with masculinity), internalizing and making their own stricter schemes for the masculine attributes than those associated with women. This greater appropriation of male roles is undoubtedly related to greater exposure and salience of more strongly stereotyped male models, present in multiple learning environments. Currently, very different models of women are shown in the media and in general in public life in a normalized manner, with women presenting traditionally more masculine characteristics such as assertiveness or leadership. However, male models remain very stereotyped and their roles have not become more flexible as has been the case with women. In this line, it seems important to work at the school and family level on an educational approach that promotes alternative masculine schemes that break the constrictions of traditional masculinity (Renold, 2004), and allow boys to identify from the first years of life with men who care for others, are affective or are dedicated to feminized professions (Swain, 2005).

With respect to professional roles, general developmental differences tend to increase the flexibility of responses (greater proportion of choices in the response “both can carry out that profession”), in line with previous research (Bigler, 1995; Banse et al., 2010) that associates the decrease in biases to the increase of cognitive abilities. However, certain differences appear in developmental trajectories when considering boys and girls separately. Thus, it is observed that stereotypes regarding male professions remain stable among girls, while they decrease slightly among boys. In the case of the assignment of female professions to women, there is also a decrease in gender stereotypes as the child’s development progresses, becoming in general the most flexible participants in this type of study. However, the developmental differences are observed to be manifested mostly among boys, stereotyping these professions with less intensity as their age increases, without observing this decrease among girls.

This would imply that, in general, older boys are more flexible in the understanding of typically male or female professions than girls, which will undoubtedly have a negative impact on girls in their future vocational and professional choices. As they get older, children seem to broaden their perceptions of their possible professional expectations. However, this greater flexibility in the workplace does not seem to have been incorporated to the same extent by girls aged 8–9, especially with regard to professions traditionally considered “male.” Assuming that cognitive development is at the base of the flexibility of gender roles in general, it is worth asking why, if this development is present in equal measure in both sexes, girls have more difficulty than their male counterpart to make gender schemes more flexible in terms of the professional world. This greater constriction of the professional area among the older female participants (associating to a greater extent certain jobs with men and women), compared to their male classmates, can be found at the base of the gender gap observed in the workplace, along with other social factors that seem to limit women’s career opportunities.

As stated in previous studies (Vervecken and Hannover, 2015), the development of vocational interests is forged in the primary education stage, so we must pay special attention to the messages that are sent from all learning environments in this regard. Furthermore, as previously mentioned, counter-stereotypical models must be offered (Olsson and Martiny, 2018) to teach children from an early age that what one dedicates their life to must be related to what one likes to do and what one does well. In this regard, it should not be forgotten that what children project as a possible profession is also influenced by the perceptions of accessibility to these jobs (status and difficulty), as well as by their own beliefs of self-efficacy (Vervecken and Hannover, 2015). In the case of the participants in this study, this perception of self-efficacy is undoubtedly diminished because, as we have seen, intelligence is associated with men in a very biased way.

### Relationship Between Variables

Regarding the relationships observed between the variables in this study, the complexity and multidimensionality of gender schemes are determined, as well as their differential application to different areas of life (Liben and Bigler, 2002d; Banse et al., 2010) and the existence of differences regarding the masculine and feminine schemes, in line with previous studies (Wilbourn and Kee, 2010; Siyanova-Chanturia et al., 2015). The correlational analysis shows a near absence of significant correlations between the measures included in the study, although the stereotyping processes analyzed are supposedly based on the common application of an underlying gender scheme (Bem, 1981, 1983; Weisgram, 2016). The only two measures that seem to correlate in a positive but moderate way when considering all the participants at a general scale are the assignment of male professions to men and female professions to women. This indicates that the participants (especially the boys) who most believe that police officers or captains should only be men, also tend to think that florists and hairdressers should always be women. However, a similar relationship is not observed in the field of personal characteristics, as one would expect if we were faced with a monolithic scheme that is applied with the same intensity to different areas. In short, gender schemes seem to be gradually incorporated into children’s development and with different intensity depending on the specific scheme that is activated (male or female), as well as their area of application, as indicated by other studies in this area (Liben and Bigler, 2002e).

Furthermore, when the relationships between variables in the different age groups were analyzed, none of the variables considered were found to be significantly related among the children. This data indicates that in these ages the gender scheme is still forming and turns out to be quite inconsistent. From this moment on, the data indicate a greater coherence between the responses, probably caused by a gradual incorporation of environmental knowledge and experiences that feed the information that is socially associated with the labels of men and women. In the group of the 6- and 7-year-old participants there is already a greater consistency among gender schemes in the professional field, which continues to increase in the group of the older participants. At 8 and 9 years of age it seems that there is already a greater consistency in the gender scheme when applied to the two areas analyzed (personal attributes and professions), but only with respect to male attributes and professions, not with respect to women.

Ultimately, these data indicate that although gender schemes are already present at the age of 4, as children grow up, they seem to be enriching these gender schemes more consistently and coherently for different domains and with regard to the man/woman dichotomy. The masculine scheme (and all that it implies regarding personal attributes and professions) seems to be more compact in these ages than the feminine scheme which seems to be more flexible and diverse. As previously mentioned (Bigler, 1995; Halim, 2016), the more advanced cognitive development that characterizes the older ages seems to promote less rigid gender schemes for femininity in all areas, but not for masculinity. In addition, the beneficial effect of cognitive development in making these schemes more flexible seems to be more efficient among boys than among girls. At this point it is important to remember that more flexible gender schemes will promote a development that is more free in terms of how to be and what professions to carry out (Trautner et al., 2005; Banse et al., 2010), promoting a better quality of life and a more adequate future physical and mental health.

### Environmental Influences in the Construction of Gender Schemes

Based on all these findings, a series of measures can be implemented in learning contexts to promote a freer and more flexible society with respect to the identity categories of men and women are proposed below. As Bem states with his theory (Bem, 1981, 1983), the fact that a social category becomes the core of a cognitive scheme is not inevitable but rather depends on the nature of the social contexts in which this category is immersed. Social categories tend to become relevant schemes if society constantly associates a specific label with different attributes, behaviors, professions, etc. In addition, the gender category becomes a relevant variable for children when different social institutions, norms and taboos are built upon it.

Learning environments that separate boys from girls (for example, segregated schools) or, on a broader level, societies that are not equal, will promote more gender schematic children than those in which being a man or woman is merely another personal characteristic, among many others. As Bem (1983) states, when a culture insists (with explicit and implicit messages) that a social category is very important at a functional level, the passive associations that children have been able to build between that category and certain human traits becomes an active scheme that is available when interpreting the reality that surrounds them. Children will apply this scheme as far as they find it helpful to predict the world around them. This author states that children will show less sex typed behaviors if, from all educational contexts, an effort is made to avoid associations that reinforce the prevailing gender scheme. For example, distributing tasks traditionally associated with one or the other should be avoided, or presenting models of biased occupations, as is often the case in textbooks. Learning environments should also promote alternative categorization schemes, in which individual differences between people stand out above intergroup differences (emphasizing variability within a group and things that are shared between people from different groups).

Furthermore, Bem (1983) argues that it does not seem enough to ignore the prevailing sexist messages in part of society, but that the school and the family should promote a critical analysis of them. This analysis should help children understand that gender roles depend on socialization and culture and have little to do with the biological differences of men and women. With this in mind, it seems necessary to discuss the origin of gender inequalities with children, reflecting on their social and historical roots and the reasons why they still endure even though societies seem more egalitarian at the formal level.

In a similar vein, the Developmental Intergroup Theory (Bigler and Liben, 2007) states that certain environmental characteristics can promote the development of more rigid stereotypes associated with a category. This occurs, for example, when perceptual discrimination between groups is exaggerated in certain environments. In this line, educational contexts where boys and girls dress differently (for example with different school uniforms), or perceptual cues, such as earrings, used to distinguish boys from girls at birth, should be avoided.

According to these authors, contexts in which attention is drawn to creating groups, labeling them or using routines in which group membership is explicitly used as the basis for school activities also produce more rigid biases. Along these lines, schools should avoid championships in which the gender category is applied to divide the groups or using children’s sex to organize the activities they practice, the classroom or the educational center. School segregation also increases the salience of this category, so it would be detrimental when promoting more egalitarian attitudes because it fosters a more dichotomous and prescriptive worldview. In addition, according to Allport’s contact theory (Allport, 1954), prejudices are reduced when people belonging to different groups meet and interact to achieve common goals. In this sense, segregated schools would be inhibiting children from establishing contacts with people “from the other group,” thus preventing the discovery of the large number of things that they probably have in common and the benefits of dealing with human diversity.

All these precautions should be especially considered when children’s cognitive abilities are still very limited (mainly in regard to classification skills) (Bigler, 1995). In this sense, the interaction of the cognitive limitations of the first years of life with very segregated contexts (in which the salience of the gender variable is very important) can lead to the development of rigid and very limiting stereotypes that already begin to determine the choices of children, their preferences or their expectations about themselves and others in these early ages. Although in later stages cognitive skills increase, the early construction of rigid schemes in the first years of life can determine different paths that involve, for example, the choice of different types of toys, the personal attributes that they will develop to adapt to social expectations, or even the type of activities they will practice and in which they will acquire higher levels of competence. Although, throughout childhood, growing cognitive skills allow children to build less rigid schemes, early environmental experiences and the limited cognitive abilities of these ages can cause very divergent initial developments that can later be difficult to reverse.

Regarding schools, although formal educational systems are trying to be more equal every day at a theoretical or legislative level, data shows that even today the leisure environments are still very differentiated for boys and girls, both in terms of the use of space and the type of objects and activities offered (Pomerleau et al., 1990). In this regard, it is appropriate to pay attention to this aspect and ensure that school environments respect an equitable and cooperative use of space, promoting activities that are not gender biased and providing toys and materials that promote active play and children’s sense of agency for both boys and girls, while also working on activities related to mutual care and cooperation between both groups.

In addition, within learning environments, there are also a number of important influences that are not so direct which have to do with the presentation of gender roles and sexist stereotypes in the media, children’s literature or toys (Wille et al., 2018). In recent years, we have also found a powerful environmental influence regarding the information that children and young people absorb from an early age through social networks and the Internet (Murnen et al., 2016). This environmental influence includes for example, the role of youtubers, or social networks such as Instagram or Facebook, that configure different worlds for the boys and girls who approach them, both with respect to the models they transmit and with respect to the information they include (advertising, topics covered, models of masculinity and femininity, etc.) (Plakoyiannaki et al., 2008).

In short, if we want to educate today’s children to be more equal and have more freedom to choose how they want to be, without the constraints associated with traditional gender roles, we must apply a comprehensive perspective that promotes the application of the gender mainstreaming approach to all institutions that educate in today’s society (Hussain et al., 2015), beyond schools. Society educates children and laws should promote family, social, educational and media-oriented policies from this cross-cutting approach that promote the reduction of sexist attitudes and individual freedom to each develop as a person, regardless of sex or identity. In this sense, all learning environments should work together to promote more egalitarian messages that do not perpetuate traditional schemes, which can be so harmful and limiting.

### Limitations and Future Works

Regarding the limitations of this study, reference should be made to the fact that different measures have been used for the two types of domains that were analyzed (personal and professional), an aspect that may have influenced the low observed relationship between the different variables. In the first task regarding personal attributes, participants were asked to choose a man or a woman as the protagonist of the story, without giving the option to answer that both could be intelligent or kind. However, in the task of assigning professions the option of responding that “both should carry out that profession” was offered. It should be explained here that the very nature of the tasks required a different response format. Thus, the first task required “forcing” the assignment of the attribute to one of the two types of targets, since the formulation of the questions forces the child to opt for a person in question. If the option “can be anyone” were given, the task would lose its meaning. In any case, in general, the participants did not have problems to assign the attributes quickly when they were told the stories, and only 5 participants sporadically responded with “do not know - do not answer.” In addition, in the task of assigning professions it made more sense to provide a third intermediate option, since a work expectation is not being applied (who do you think is the police or the hairdresser, in which case it would be more logical to apply a dichotomous response scheme like in the previous task), but a more attitudinal response (who do you think should do that job). In any case, the flexibility that the task of professions brings could also be indirectly reflected in the subjects’ responses to the different attributes that are presented in the task, although it is not a proper response option in each test.

Furthermore, we cannot ignore that the task regarding professional stereotypes is more explicit and probably because of this it is easier for the answers to be more biased by social desirability, which can promote flexible responses. The characteristics of the task of assigning personal characteristics (forcing an answer and presenting the question implicitly) give rise to a greater projection of the schemes present in the cognitive system of children, without being aware that they are being explicitly asked about this topic.

In the face of future studies, designing measures that are more comparable to each other should be explored, allowing the collection of similar data on the different domains to which gender schemes apply, as previous studies suggest (Liben and Bigler, 2002b). In addition, it would be interesting to include measures related to the development of gender identity in these studies, as numerous studies indicate that the acquisition of these schemes is carried out in parallel and the development of the self-concept seems to play a fundamental role in this process (Martin and Ruble, 2004; Tobin et al., 2010). Ideally, replication studies should be conducted in the future with bigger samples, including higher and more balanced number of participants in each age group in order to safeguard the confidence in the developmental findings and the generalization of the results. In future research it would also be interesting to include a greater variety in regard to the type of participants, including people with diverse backgrounds and environments (for example, children of families with different socioeconomic backgrounds or parents with different type of professions, as well as students from mixed schools versus segregated schools). Those correlational studies might be helpful to improve our knowledge on the influence of environmental variables on acquisition and development of gender schemes. In this line, from an experimental approach, it would be interesting to apply intervention models that modify some of the aspects of the environment previously mentioned (for example, the presence of women in textbooks, or the development of more inclusive schoolyards) to be able to observe the effect of these environmental modifications on the formation of gender stereotypes.

Finally, it seems necessary to cover a broader range of children’s ages in this type of study, given that the results observed in the group of the oldest children continue to show a wide presence of gender biases in the two analyzed areas (especially in terms of personal attributes and in the case of the masculine scheme), although the cognitive abilities of children in these ages already allow them to move toward more flexible schemes.

## Data Availability Statement

The datasets generated for this study are available on request to the corresponding author.

## Ethics Statement

Ethical review and approval was not required for the study on human participants in accordance with the local legislation and institutional requirements. Written informed consent to participate in this study was provided by the participants’ legal guardian/next of kin.

## Author Contributions

IS-C, SV-M, and PH-H have equally participated in all the tasks carried out to conclude this research and the paper itself: search of references, design of the material, data collection, data analysis, and writing of the manuscript.

## Conflict of Interest

The authors declare that the research was conducted in the absence of any commercial or financial relationships that could be construed as a potential conflict of interest.
