# Supplementary material for: Socialization of Gender Stereotypes Related to Attributes and Professions Among Young Spanish School-Aged Children
Source: Front Psychol. 2020 Apr 24;11:609. doi: 10.3389/fpsyg.2020.00609 (PMC7194082; doi:10.3389/fpsyg.2020.00609)
Supplement: Supplementary file 1 [file Data_Sheet_1.pdf]

Supplementary Material

1 Supplementary Data

GENDER STEREOTYPE TASK INSTRUCTIONS.

TASK 1. PERSONAL ATTRIBUTES.

The task is to tell each participant a story, and then ask who they think the protagonist of that story is. The story can be about an adult or a child. The stories are based on the study of Bian, Cimpian and Leslie (2017).

Task 1. Adult targets (A)

Begin by introducing and greeting the child. Say "I'm going to tell you what some people I work with are like".

Smart

In the place where I work there are many people. But there is a person who is different from the others. That person is very, very clever. That person does things faster and better than others. That person is really quite smart. Who do you think that person is?

Happy

In the place where I work there are many people. But there is a person who is different. That person is very, very happy. That person laughs a lot and likes to have fun. This person is really quite happy. Who do you think that person is?

Kind

In the place where I work there is another different person. That person is very, very kind. That person likes to help others with their problems and is very nice to everyone in the office. This person is really quite kind. Who do you think that person is?

Aggressive

In the place where I work there are many people. But there is a person who is different. That person behaves very badly. This person argues with others and insults them when they don't do what the person wants. This person is really aggressive, (is someone aggressive). Who do you think that person is?

Vain

In the place where I work there are many people. But there is a person who is different. That person is very, very vain. This person looks a lot in the mirror and worries about whether their hair and clothes look good. This person is really quite vain. Who do you think that person is?

Grumpy

In the place where I work there are many people. But there is a person who is different. That person is always in a bad mood. This person gets very angry and complains about everything. This person is quite grumpy. Who do you think that person is?

Task 1. Children targets (B)

Start by saying: "Now I am going to tell you some stories of what some children were like when I was in school."

Smart

When I was your age I went to a school where there were many children. But there was someone who was different, because this child was very very clever. This child learned things very quickly and answered all the teacher's questions. This child was very, very smart. Who do you think it was?

Happy

When I was your age I went to a school where there were many children. But there was someone who was different. This child was very, very happy. This child laughed a lot and liked to have fun. The child was really quite happy. Who do you think it was?

Kind

When I was your age I went to a school where there were many children. But there was someone who was different, this child was very, very kind. This child shared toys with all the children and was careful with others. This child was very, very kind. Who do you think it was?

Aggressive

When I was your age I went to a school where there were many children. But there was someone who was different. This child behaved very badly, argued with others and insulted them when they didn't do what the child wanted. This child was someone very aggressive, a very aggressive child. Who do you think it was?

Vain

When I was your age I went to a school where there were many children. But there was someone who was different. This was very, very vain. This child looked a lot in the mirror and worried about whether their hair and clothes looked good. This child was very vain. Who do you think it was?

Grumpy

When I was your age I went to a school where there were many children. But there was someone who was different. This child was always in a bad mood. This child would get very angry and complain about everything. This child was quite grumpy. Who do you think it was?

TASK 2. PROFESSIONAL ROLES.

This task is based on the instrument designed by Liben and Bigler (2002). After completing Task 1, the child is told that we are now going to play a card game called “Who should do this job?”. The following is explained: “I will show you cards that relate to different jobs and you have to do the following: if you think that this job should only be done by men you have to put the card here, in this box where there is a picture of a man. If you think that this job should only be done by women, put it here, where there is a picture of a woman. And if you think men and women should do it, put it here.” After the explanation, the child will be shown the different cards and asked who they think should be ...

the person who gives you the injection when you go to the doctor? (NURSE)

the person who directs the traffic? (POLICE)

the person who is at the checkout when we go to the supermarket? (CASHIER)

the person who does paintings? (ARTIST)

the person who leads a ship? (SHIP CAPTAIN)

the person who arranges the flowers at the flower shop? (FLORIST)

the person using the microscope to find viruses? (SCIENTIST)

the person who makes bread? (BAKER)

the person who cuts hair at the hairdresser? (HAIRDRESSER)

the person who fixes the computers when they don’t work? (COMPUTER SPECIALIST)

Bian L, Leslie SJ, Cimpian A. Gender stereotypes about intellectual ability emerge early and influence children’s interests. Science. 2017;355(6323):389–91.

Liben LS, Bigler RS. I . Introduction. Monogr Soc Res Child Dev. 2002;67(2):1–7.

Liben LS, Bigler RS. V . Empirical Data Related To Developmental Pathways. Monogr Soc Res Child Dev. 2002;67(2):76–95.

Liben LS, Bigler RS. IV. Empirical Data Related to Scale Development. Monogr Soc Res Child Dev. 2002;67(2):40–75.

Liben LS, Bigler RS. III. Gender Constructivism Reconsidered. Monogr Soc Res Child Dev. 2002;67(2):22–39.
